# Association of Newly Found Asymptomatic Intracranial Artery Stenosis and Ideal Cardiovascular Health Metrics in Chinese Community Population

**DOI:** 10.1038/s41598-020-63927-3

**Published:** 2020-04-29

**Authors:** Changfeng Fan, Qian Zhang, Shufeng Zhang, Anxin Wang, Xinwei Bi, Shengyun Chen, Zhaoxia Li, Shouling Wu, Xingquan Zhao

**Affiliations:** 1 Department of Neurology, Beijing Tiantan Hospital, Capital Medical University, Beijing, 100050 China; 2 Department of Neurology, Peking University Shougang Hospital, Beijing, 100144 China; 3 Department of Neurology, the General Hospital of Chinese People’s Armed Police Forces, Beijing, 100142 China; 4 Department of Cardiology, Kailuan Hospital, Tangshan, 063000 China

**Keywords:** Lifestyle modification, Stroke

## Abstract

In the general population, there is a strong inverse relationship between the number of ideal cardiovascular health (CVH) metrics and the total incidence of cardiovascular diseases and stroke. However, the prevalence of ideal CVH is extremely low and there are few studies on its association with newly found asymptomatic intracranial arterial stenosis (AICAS). Therefore, we performed this prospective study to assess the relationship between the newly found AICAS and ideal CVH metrics in the Chinese community population. Seven ideal CVH metrics of 3,475 participants in the Asymptomatic Polyvascular Abnormalities Community study (APAC) conducted in China (1,962 men and 1,513 women between the ages of 45 and 75 years) were collected. Based on the occurrence of newly found AICAS, all participants were divided into the AICAS group and non-ICAS group. Prevalence of ideal CVH metrics was compared between the two groups. Logistic regression was used to estimate the association of newly found AICAS with ideal CVH metrics. The result was the number of ideal CVH metrics was strongly associated with age, gender, education levels and family income (each *P* < 0.0001). Among the seven CVH metrics total cholesterol (TC) was the only one showing significant difference between the newly found AICAS group and non-ICAS group in our 2 years observation. Participants with less ideal CVH metrics (≤3) were associated with significantly higher prevalence of AICAS than those with more (>3) ideal CVH metrics (*OR*, 1.27; *P* = 0.045). Furthermore, less (≤3) ideal CVH metrics was markedly associated with higher incidence of AICAS for all participants, younger participants (<60 years) (*OR*, 1.34; *P* = 0.046) and men participants (*OR*, 1.53; *P* = 0.032) after adjustment for gender, age, education level, family income and stroke history. Thus we conclude that participants with newly found AICAS have high prevalence of total cholesterol status, and Individuals with low ideal CVH metrics (≤3) are associated with significantly higher prevalence of asymptomatic ICAS, especially in high-risk population of young and men participants. Therefore, primordial prevention of stroke should also focus on those high-risk populations.

## Introduction

Studies have confirmed that intracranial artery stenosis (ICAS) was one of the most common causes of stroke^1–3^, accounting for nearly 10.0% of all cerebral ischemic events^4,5^. Meanwhile, Population-based studies have shown that ICAS was found commonly among stroke patients of Asians, Blacks, and Hispanics ancestry^2,3^, and even under the best medical therapy symptomatic ICAS-related stroke recurrence continues to be the highest among all stroke etiologic subtypes. Therefore, primordial prevention of stroke through risk factors reduction is fundamental. AICAS is a chronic and progressive disease until it becomes symptomatic, which leads to stroke and the patient will be subject to high risk of stroke recurrence and other vascular events such as coronal or limb infracts, and life-long therapy and disability. The heavy financial burden incurred thereafter, will affect their and their family’s life quality^6^. Thus enhancing our knowledge about AICAS especially its risk factors can help reduce its disastrous clinical consequence.

The American Heart Association (AHA) defines a simplified seven-item tool including four ideal health behaviors (nonsmoking, body mass index <25 kg/m^2^, physical activity at goal levels, and a healthy diet) and three ideal health factors (normal cholesterol <200 mg/dL, blood pressure <120/<80 mm Hg, and fasting blood glucose <100 mg/dL) as a complimentary option for primordial prevention of cardiovascular disease (CVD) in community population^7^. However, the prevalence of ideal CVH is extremely low in American, European and Asian populations^8,9^. Hence, individualized prescription and minimum targeted number of ideal CVH metrics that can be easily achieved should be explored.

Previous studies in this area are generally of cross-sectional design, and there is possible bias as some individuals might have AICAS already and the ideal CVH metrics parameters have changed. No study has evaluated the minimum targeted number of ideal CVH metrics to be adopted for the primordial prevention of ICAS and few studies evaluated the association between newly found asymptomatic intracranial arterial stenosis and CVH metrics in community population. Therefore, we have performed this prospective study to analyze the effect of every ideal CVH metrics on AICAS, assess the minimum number of ideal CVH metrics to predict AICAS, and then provide reliable evidences to screen vulnerable population delay the development of AICAS and achieve primordial prevention of stroke.

## Results

There were 3,475 participants in the APAC study. We identified 352 newly found ICAS (10.12%) among 1,513 women and 1,962 men.

Basic characteristics were shown in Table 1. Moreover, the number of CVH was significantly associated with the gender (P < 0.0001), age (P = 0.001), education levels (P < 0.0001) and also the income of the family (P < 0.0001); but there was no statistic relationship between number of CVH and family history of stroke (P = 0.3999) (Table 1).

**Table 1:** Basic characteristics according to the number of ideal CVH metrics.

	Number of Ideal cardiovascular health metrics							
	0	1	2	3	4	5	6 or 7	*P* value
**Basic**								
Women(n,%)	1(1.43)	53(17.49)	181(25.78)	364(40.44)	454(54.37)	323(66.54)	137(76.54)	<0.0001
Age M ± SD(y)	51.23 ± 8.70	53.09 ± 9.31	53.63 ± 10.07	53.79 ± 10.35	53.95 ± 11.15	52.99 ± 11.42	51.37 ± 11.45	0.0001
**Education(n,%)**								
Illiteracy/primary	10(14.29)	41(13.53)	75(10.68)	95(10.56)	69(8.26)	33(6.79)	7(3.91)	<0.0001
Middle school	30(42.86)	148(48.84)	331(47.15)	419(46.56)	345(41.32)	156(32.10)	60(33.52)	<0.0001
High school or above	30(42.86)	114(37.62)	296(42.17)	386(42.89)	421(50.42)	297(61.11)	112(62.57)	<0.0001
**Income (n,%)**								
<¥ 1000	19(27.14)	72(23.76)	177(25.21)	212(23.56)	185(22.16)	113(23.25)	40(22.35)	<0.0001
¥ 1000–3000	48(68.57)	207(68.32)	460(65.53)	603(67,00)	554(66.35)	321(66.05)	120(67.04)	<0.0001
≥ ¥ 3000	3(4.29)	24(7.92)	65(9.26)	85(9.44)	96(11.50)	52(10.70)	19(10.61)	<0.0001
**Previous history of disease(n,%)**								
Diabetes	20(28.57)	70(23.10)	121(17.24)	85(9.44)	49(5.87)	5(1.03)	1(0.56)	<0.0001
Hypertension	41(58.57)	194(64.03)	412(58.69)	424(47.11)	306(36.65)	91(18.72)	20(11.17)	<0.0001
Dyslipidemia	64(91.43)	236(77.89)	420(59.83)	424(47.11)	290(34.73)	107(22.02)	32(17.88)	<0.0001
Family history of stroke	2(2.86)	9(2.97)	21(2.99)	43(4.78)	26(3.11)	14(2.88)	7(3.91)	0.3999

Based on the occurrence of newly found AICAS, all participants were subdivided into the AICAS group (n = 352) and non-ICAS group (n = 3123). Gender (P < 0.0001), age (P = 0.003), education level (P = 0.0006), and the history of diabetes (P = 0.004) or dyslipidemia (P = 0.02) showed a notable difference between the two groups, while family income levels (P = 0.06) and the rates of history of hypertension (P = 0.41) were similar between the two groups (Table 2).

**Table 2:** Characteristics of participants with or without AICAS.

	AICAS	non-ICAS	*P* value
Total(n,%)	352(10.13)	3123(89.87)	
Women(n,%)	197(55.97)	1316(42.14)	<0.0001
Mean age ±SD(y)	55.21 ± 11.37	53.25 ± 10.49	0.003
**Education (n,%)**			
Illiteracy/primary	27(7.67)	303(9.70)	**0.0006**
Middle school	123(34.94)	1366(43.74)	
High school or above	202(57.39)	1454(46.56)	
**Income (n,%)**			
<¥ 1,000	748(23.95)	70(19.89)	0.0624
¥ 1,000–3,000	2076(66.47)	237(67.33)	
≥¥ 3,000	299(9.57)	45(12.78)	
**Previous history of disease**			
Diabetes (n,%)	51(14.49)	300(9.61)	**0.004**
Hypertension (n,%)	158(44.89)	1330(44.60)	0.4086
Dyslipidemia (n,%)	180(51.14)	1393(44.60)	**0.0196**
Family history of stroke (n,%)	15(4.26)	107(3.43)	0.4196

All participants were divided into two groups according to whether they had AICAS, and significant difference was only found in TC (P = 0.002), other factors did not differ significantly between the two groups (Table 3). Further analysis of the relationship between different number of CVH metrics and AICAS showed number 3 had the biggest Youden’s index (0.028109). Thus, based on the number of ideal CVH metrics, all participants were divided into less ideal CVH metrics group (≤3) (n = 1975) and more ideal CVH metrics group (>3) (n = 1500). The incidence of AICAS was not significantly different in all patients, irrespective of age and gender (all P > 0.05). However, after adjustment of the gender, age, education level, family income and stroke history, participants with less number of ideal CVH metrics (≤3) was markedly associated with higher incidence of ICAS (OR, 1.27; 95%CI, 1.01–1.61; P = 0.045), especially in younger patients (<60 years) (OR, 1.34; 95% CI, 1.00–1.78; P = 0.046) and male participants (OR, 1.53; 95% CI, 1.04–2.25; P = 0.03). Although not reaching the P = 0.05 (P = 0.055) threshold, the relationship between AICAS and CVH metrics in the older subset (OR, 1.53; 95% CI, 0.99–2.37; P = 0.055) was at borderline, given the high odds ratio of 1.53 compared to1.34 in younger persons. There were no significant association between the number of ideal CVH metrics and AICAS in women participants (OR, 1.29; 95% CI, 0.94–1.76; P = 0. 11) (Table 4).

**Table 3:** Prevalence of health behaviors or factors (ideal, yes/no) in participants regarding whether they have AICAS in 2012.

Variables	Total	AICAS	non-ICAS	P value
**Smoking**				
Ideal (%, n)	63.68 (2213)	9.35 (207)	90.64(2006)	
Non-ideal (%, n)	36.32 (1262)	8.32(105)	91.68(1157)	0.4532
**Body mass index**				
Ideal (%, n)	54.24(1885)	10.29(194)	89.71(1691)	
Non-ideal (%, n)	45.76(1590)	9.94(158)	90.06(1432)	0.7353
**Physical activity**				
Ideal (%, n)	33.93(1179)	0.86(128)	89.14(1051)	
Non-ideal (%, n)	66.07(2296)	9.76(224)	90.24(2072)	0.3132
**Diet**				
Ideal (%, n)	20.81(723)	9.96(72)	90.04(651)	
Non-ideal (%, n)	79.19(2752)	10.17(280)	89.83(2472)	0.8901
**Fasting blood glucose**				
Ideal (%, n)	70.47(2449)	10.21(250)	89.79(2199)	
Non-ideal (%, n)	29.53(1026)	9.94(102)	90.06(924)	0.8534
**Total cholesterol**				
Ideal l (%, n)	59.77(2077)	8.81(183)	91.19(1894)	
Non-ideal (%, n)	40.23(1398)	12.09(169)	87.91(1229)	0.0019
**Blood pressure**				
Ideal (%, n)	21.17(661)	11.63(87)	88.37(661)	
Non-ideal (%, n)	78.83(2462)	9.72(265)	90.28(2462)	0.1323

**Table 4:** Odds ratios for AICAS in two groups with low and high levels of ideal CVH metrics.

3475 cases	Number of ideal CVH metrics		*P* value
	Low (≤3)	High (<3)	
**Total**			
Participants(n)	1975	1500	
Case (%)	56.83	43.17	
Crude OR (95%CI)	0.99 (0.80–1.25)	1	0.994
Adjusted OR(95%CI)	1.27 (1.01–1.61)	1	0.045
**<60** (**year**)			
Participants(n)	1550	1153	
Case (%)	44.60	33.18	
Crude OR (95%CI)	0.88 (0.68–1.15)	1	0.353
Adjusted OR (95%CI)	1.34 (1.00–1.78)	1	0.046
**≥60** (**year**)			
Participants (n)	425	347	
Case (%)	12.23	9.99	
Crude OR (95%CI)	1.41 (0.92–2.17)	1	0.113
Adjusted OR (95%CI)	1.53 (0.99–2.37)	1	0.055
**Men**			
Participants (n)	1376	586	
Case (%)	39.60	16.86	
Crude OR (95%CI)	1.16 (0.80–1.68)	1	0.432
Adjusted OR (95%CI)	1.53 (1.04–2.25)	1	0.032
**Women**			
Participants (n)	599	914	
Case (%)	17.24	26.41	
Crude OR (95%CI)	1.24 (0.92–1.68)	1	0.159
Adjusted OR (95%CI)	1.29 (0.94–1.76)	1	0.111

## Discussion

Number of ideal cardiovascular health (CVH) metrics was in significantly negative/inverse association with the total incidence of cardiovascular diseases and stroke for high-risk participants^7–9^. Our previous studies confirmed that high number of ideal CVH metrics was markedly associated with a lower rate of stroke (total, ischemic, and hemorrhagic) and ICAS in the Chinese population^8,9^. Therefore, a bigger number of ideal CVH metrics was associated with a lower incidence of CVD and non-CVD outcomes. Based on the analysis of basic characteristics in the AICAS participants, it was found that the number of ideal CVH metrics was significantly associated with gender, age, education levels, family income, and history of diabetes, hypertension, dyslipidemia, but no relation with family history of stroke (P = 0.4). Those participants, the female, the young in age, those of higher family income and good education tended to have bigger number of ideal CVH metrics but only 5.15% of the total participants fall in the group of biggest number 6–7 while 56.83% participants achieve three or less. The findings are similar to our previous studies, and the distribution is similar to US or non-US populations in other studies^10,11^. The prevalence of high number of ideal CVH metrics is extremely low. In the U.S., the prevalence of 6 to 7 ideal CVH metrics was as low as 0.5% for African-Americans^12^. In one cohort population of China, only 7 of 15,350 participants (0.05%) met all 7 ideal CVH metrics^9^. Therefore, individualized actions should be taken to obtain the easily achievable number of ideal CVH metrics^13,14^. Overall, for the general population, the prevalence of ideal classification for smoking is less than 50%, body weight less than 40%, physical activity less than 30%, blood cholesterol less than 40%, diet less than 10%, blood pressure less than 20%, and blood glucose less than 60%^8^. In our study, it reads 63.68%, 54.24%, 33.93%, 20.81%, 70.47%, 59.77% and 21.17% respectively. Therefore, the highest ideal metrics were smoking and blood glucose and the lower ideal metrics were diet, physical activity, and blood pressure metrics. Furthermore, participants with no more than three ideal CVH metrics were associated with significantly increased risk of ICAS, and they are the key population for primordial prevention of stroke. Hence, for the high-risk population, individualized strategies are needed to improve the CVH comprehensive score, so as to decrease the risk of ICAS and stroke.

Among all ideal CVH metrics, only TC showed significant difference (P = 0.0019) for AICAS group and non-ICAS group. Dyslipidemia has been proven to be strongly associated with the severity of AICAS, and it is one of the most common risk factors related to severe ICAS^15,16^. Animal model tests have also confirmed the serious effects of lipids on arteriosclerosis–Yamori Y *et al*. successfully produced fat deposition in the posterior communicating arteries of normotensive rats after 10 weeks of feeding with high-fat cholesterol^17^. Our previous study showed that TC level was an independent risk factor for the occurrence of AICAS^17^. By investigation on a group of 1,471 patients with TIA or minor stroke, Sirimarco G *et al*. revealed that the risk of early stroke recurrence was significantly higher in the patients with hyperlipidemia than those without^16^. And a study showed that by lipid-lowering therapy, 90-day vascular event risk in non-disabling ischemic cerebrovascular disease could be reduced by 35–40% approximately^18^. Though our follow-up period was only 2 years, the significant effect of TC on AICAS was apparent. Thus, it is seriously urgent to keep an ideal TC level in the whole lifetime, not only after or before the stroke attack.

Though other CVH metrics such as smoking, FBG, blood pressure, BMI, PA and diet were supposed to account for ICAS, and in our study they displayed difference between AICAS and non-ICAS groups, but the difference had no significant statistic meaning. It may have something to do with the short follow-up period so a longer observation is needed to find their effect on the slow progressing change of artery stenosis. In addition, the accuracy of TCD to detect ICAS especially its morphological changes are limited.

Our previous cross-sectional study had showed the inverse relationship between the number of ideal CVH metrics and AICAS^11^. This prospective study also displayed similar findings. A smaller number of ideal CVH metrics (≤3) was markedly associated with higher incidence of AICAS, especially in younger and male participants.

Younger participants are commonly healthier and have less risk factors of cardiac and cerebrovascular disease. But those who have a low number of CVH metrics tended to have AICAS, therefore, to prevent vascular disease, such as stroke, the number of ideal CVH metrics should be maximally enlarged by keeping good life habits and controlling blood pressure, cholesterol and triglyceride levels, and diabetes mellitus especially for young and male participants. However, these findings need to be further verified with more studies in different populations.

Females are likely to have more ideal CVH metrics. Several studies have indicated a sex disparity in ideal CVH metrics^19,20^. In our study, only 54 women, or 1.53% of the sample, have no or one CVH metrics. Simon *et al*. have showed that women were four times more often in ideal CVH and two times more often in intermediate CVH than men^20^, though this sex disparity in ideal CVH metrics decreased with age and disappeared after the age of 65^19,20^. Several reasons are behind this sex disparity in ideal CVH metrics, e.g. women obtained more primary care and preventive services than men^21^. The EUROASPIRE IV study showed that more women with coronary artery disease attempted to eat healthier food and to quit smoking than men^22^. Meanwhile, previous clinical trials demonstrated that the progression of intracranial arterial stenosis was different between female and male. Furthermore, sex hormone might also play a role. Estrogen could enhance NO-mediated relaxation in the intracranial artery, reduce NADPH-oxidase activity and prolong the effects on cardiac and cerebrovascular systems^22–25^. Those over 60 years old, with the increase of age and concomitant diseases, factors contribute to ICAS also increase. Previous studies have acknowledged that Asian populations showed increased diffuse intracranial and extracranial stenosis with age, while young people showed increased intracranial stenosis^26^. So, for old population the examination of both intracranial and extracranial arteries stenosis may give a better judgement of the progress. And the association between CVH metrics and AICAS in older adults (>60 years) and women, not reaching a p below 0.05, may have relation to their smaller sample sizes.

Our study found that keeping not more than 3 ideal CVH metrics could significantly increase the incidence of AICAS. The significance was greater (P = 0.03) for men under 60 s. Therefore, the total number of ideal CVH metrics was related with AICAS for young and men participants. Great progression was found in those who have no more than 3 ideal AHA CVH metrics. These relationships need to be investigated with more studies, especially on large community and different races (populations). This study is the first time to report the minimum ideal CVH metrics number and show the importance of individualized prevention of AICAS. This can give strong support to social health care and health education, guide our population to keep health lifestyle, keep ideal level of TC, blood glucose and blood pressure. It will help prevent the progressing of AICAS and reduce the occurrence of stroke, so we call it the primordial prevention of stroke.

We identified 352 newly found ICAS (10.12%), subjects without good temporal windows are considered no stenosis. Though TCD has irreplaceable advantages in community population survey, there are some people who have poor penetration of the temporal window, leading to temporal bone window failure (WF). The incidence of WF reported in different studies fluctuates between 5–30%^27–32^. The WF in our study is 4.1%, the ideal result is related to the following reasons. Firstly, age is an important influencing factor^31,32^, the average age of our community population is relatively young. Secondly, multiple studies, including ours, have found that women are more likely to develop WF, especially in postmenopausal women^28,31^. The proportion of men in our population is significantly higher than that of women. Lastly, except for age and sex, hyperlipidemia^29^ and high body mass index^31^ are also risk factors for WF. The WF rate of community population should be significantly lower than that of cerebrovascular patients who commonly have hyperlipidemia and/or high body mass index.

### Limitations

There were several limitations in our study. First, our study is not a nationwide study, and lots of participants are manual workers, including coalminers. Therefore, there is a risk of selection bias and our findings cannot directly be generalized to other populations. Second, the validated dietary and physical activity questionnaires are not be used and we used the salt intake as a surrogate of diet quality, which is a risk factor of stroke and is a serious situation in China^33,34^. However, other aspects, including consumption of carbohydrates, protein, fat and fruit/vegetables should also be considered. Therefore, our findings should be interpreted with caution. Third, ICAS is diagnosed by TCD, possibly with high negative predictive value to screen intracranial occlusive disease in large-scale general populations^35,36^. Subjects without good temporal windows are considered no stenosis. There is no confirmation of MR angiography or other forms of angiography. Fourth, basic information was collected through questionnaires, and the investigators did not conduct preventive education on cardio-cerebral vascular disease, nor did they recommend and follow-up testing of the medication including statins. Each questionnaire only asks about the medication situation in the past 3 months. Therefore, in our final statistical analysis, we cannot analyze statins as a valid factor. Fifth, the follow-up period was 2 years, while AICAS is a relatively slow process; a longer follow-up period may strengthen our findings.

## Conclusion

TC is the most sensitive among the seven CVH metrics, which has the most important role in the progressing of AICAS in a short period. So keep an ideal level of TC all the time should be important to reduce the occurrence and progressing of AICAS. Participants with no more than three CVH metrics are associated significantly with higher prevalence of AICAS, especially for younger participants and men participants. They are the key population of primordial prevention for stroke by enlarge the number of CVH metrics to have a more healthy life-style.

## Methods

### Study Design and participants

**Figure 1:**
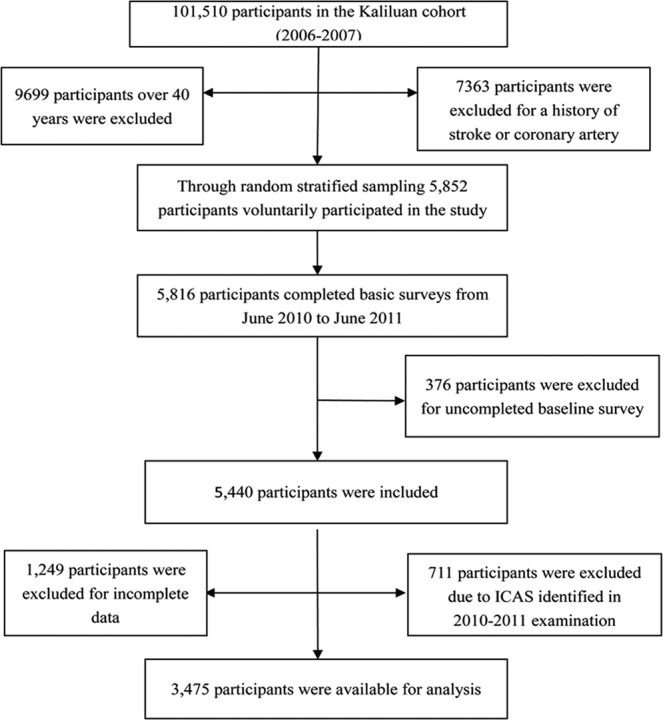
Flow diagram.

The Asymptomatic Polyvascular Abnormalities Community study (APAC), based on the Kailuan community, is a prospective cohort study in Tangshan City, southeast of Beijing, aimed to explore the epidemiology of peripheral artery diseases carotid atherosclerosis and asymptomatic ICAS in Chinese adults. 5,852 participants were randomly sampled from an ongoing prospective study in Kailuan community in Tangshan city, known as Kailuan study, which began from June 2006, and the total population was 101,510, aged 18–98 years^10^. The inclusion criteria were: (1) age over or equal to 40 years; and (2) no coronary artery disease, transient ischemic attack, and stroke. 376 participants were excluded for uncompleted baseline survey, and there were 5,440 participants left in the APAC study. All the participants, from June 2010 to June 2011, were surveyed by a questionnaire and received the examinations of physical checking, laboratory testing and transcranial Doppler (TCD) 711 participants were eliminated who had ICAS in TCD examinations between June 2010 and June 2011; therefore, 4,729 participants without ICAS were initially chosen. However, 1,254 cases were excluded for incomplete data about health factors, behaviors or absence TCD results. Finally, 3,475 cases, who had undergone TCD examination in 2012–2013, were included in the APAC study (Fig. 1). The study was performed in accordance with the guidelines of the Helsinki Declaration and was approved by the Ethics Committees of the Kailuan General Hospital and Beijing Tiantan Hospital. Written informed consents were obtained from all participants.

### Basic characteristics

Questionnaires, which were designed at the beginning of our study, as recommended in our previous studies^11,12^ were used to collect demographic variables. The family income of per month was categorized as “<1,000 ¥”, “1,000–3,000 ¥” or “≥3,000 ¥”. Meanwhile the education levels were reported as “high school or higher”, “middle school” or “illiteracy or primary school”. Meanwhile, other information included stroke history of immediate family members, smoking, physical activity and dietary intake was collected by questionnaires investigation. Smoking habits was divided into “never”, “former”, or “current”. Dietary data were is defined as three categories: “low” means the salt intake was under 6 g/day, and “medium” with a definition of 6–10 g/day, “high” as over 10 g/day. Physical activities were also divided into three subgroups as “very active (more than 80 minutes per week)”, “moderately active (less than 80 minutes per week)”, or “inactive (none)”. Moreover, Weight and height were measured and body mass index (BMI) was calculated as weight (kg)/height (m)^2^.

Blood pressure was assessed at least two times using an appropriate cuff size after the participant had a rest in a chair for at least five minutes. Two readings of systolic blood pressure (SBP) and diastolic blood pressure (DBP) were taken at a five-minute interval. Hypertension was defined as one of the below situations: there was a previous history of hypertension; currently taking hypotensive therapy; SBP was over or equal to 140 mmHg or DBP over or equal to 90 mmHg.

An overnight blood sample of each participant was collected to measure the fasting blood glucose level, the cholesterol and triglyceride levels. Grouping and definition of diabetes hyperlipidemia, BMI, blood pressure, fasting blood glucose and total cholesterol were described previously with minor modifications^11^. Diabetes mellitus was defined as a self-reported history or currently treated with insulin or oral hypoglycemic agents, or fasting blood glucose level ≥126 mg/dl. Hyperlipidemia was defined as a self-reported history, current use of cholesterol lowering medicine, or total cholesterol level ≥220 mg/dl or triglyceride ≥150 mg/dl. BMI was divided into ideal (<25 kg/m^2^), intermediate (25 to 29.9 kg/m^2^) or poor (≥30 kg/m^2^). Meanwhile, blood pressure was divided into ideal (SBP < 120 mm Hg and DBP < 80 mm Hg and untreated), intermediate (120 mm Hg≤SBP ≤ 139 mm Hg, 80 mm Hg≤ DBP ≤ 89 mm Hg, or treated to SBP/DBP < 120/80 mm Hg), or poor (SBP ≥ 140 mm Hg, DBP ≥ 90 mm Hg, or treated to SBP/DBP > 120/80 mm Hg). Moreover, fasting blood glucose was divided into ideal (<100 mg/dL and untreated), intermediate (100 to 125 mg/dL or treated to <100 mg/dL), or poor (≥126 mg/dL or treated to ≥100 mg/dL); and total cholesterol status was divided into ideal (<200 mg/dL and untreated), intermediate (200 to 239 mg/dL or treated to <200 mg/dL), or poor (≥240 mg/dL or treated to ≥200 mg/dL), respectively. We dichotomized each health metric as 0 or 1: “ideal” was coded as 1 and “non-ideal” (“intermediate” or “poor” categories) as 0. The participants’ age was classified into two categories: 40–59 years and ≥ 60 years.

### TCD examination and criteria of ICAS

TCD examinations were performed by two experienced neurologists blinded to the baseline information of all participants (Nicolet/EME Company, Germany). Meanwhile, stenotic arteries were diagnosed according to the peak systolic flow velocity of the examined artery: >140 cm per second for the middle cerebral artery, >120 cm per second for the anterior cerebral artery, >100 cm per second for the posterior cerebral artery and vertebra-basilar artery, and >120 cm per second for the siphon internal carotid artery^10,11^. Subjects without good temporal windows were considered non-stenosis^37^.

### Statistical Analyses

Continuous variables were described by mean (standard deviation [SD]) and compared with ANOVA analysis. Categorical variables were described by percentages and compared using Chi Square tests. Few subjects were in the subgroup of 6 or 7 ideal CVH metrics, so the subgroups of 6/7 ideal metrics were combined together. Prevalence of ideal CVH metrics were compared between those with or without newly found AICAS. We used youden’s index to find the number of ideal CVH metrics to predict the risk of newly found AICAS. Moreover, we used the Logistic regression analyses to evaluate the association of ICAS across health metric categories by calculating the odds ratios (ORs) and 95% confidence interval (CI). Furthermore, several parameters including age, sex, education level, income and family history of stroke are known or possible risk factors for ICAS in previous studies^14,38,39^, therefore we adjusted for these parameters in the models. All statistical tests were two-sided, and a 0.05 significance level was set. All statistical analyses were performed with SAS software, version 9.1 (SAS Institute, Cary, North Carolina, USA).
